# Nuclear Lipid Microdomains Regulate Daunorubicin Resistance in Hepatoma Cells

**DOI:** 10.3390/ijms19113424

**Published:** 2018-11-01

**Authors:** Michela Codini, Carmela Conte, Samuela Cataldi, Cataldo Arcuri, Andrea Lazzarini, Maria Rachele Ceccarini, Federica Patria, Alessandro Floridi, Carmen Mecca, Francesco Saverio Ambesi-Impiombato, Tommaso Beccari, Francesco Curcio, Elisabetta Albi

**Affiliations:** 1 Department of Pharmaceutical Sciences, University of Perugia, 06126 Perugia, Italy; michela.codini@unipg.it (M.C.); carmela.conte@unipg.it (C.C.); samuelacataldi@libero.it (S.C.); mariarachele.ceccarini@unipg.it (M.R.C.); patriafederica@gmail.com (F.P.); tommaso.beccari@unipg.it (T.B.); 2 Department of Experimental Medicine, University of Perugia, 06126 Perugia, Italy; cataldo.arcuri@unipg.it (C.A.); carmen.mecca@studenti.unipg.it (C.M.); 3 Laboratory of Nuclear Lipid BioPathology, CRABiON, 06122 Perugia, Italy; andrylazza@gmail.com (A.L.); info@crabion.it (A.F.); 4 Dipartimento di Area Medica, University of Udine, 33100 Udine, Italy; ambesis@me.com (F.S.A.-I.); francesco.curcio@uniud.it (F.C.)

**Keywords:** sphingomyelin, cholesterol, nuclear membrane, nuclear lipid microdomains, daunorubicin

## Abstract

Daunorubicin is an anticancer drug, and cholesterol is involved in cancer progression, but their relationship has not been defined. In this study, we developed a novel experimental model that utilizes daunorubicin, cholesterol, and daunorubicin plus cholesterol in the same cells (H35) to search for the role of nuclear lipid microdomains, rich in cholesterol and sphingomyelin, in drug resistance. We find that the daunorubicin induces perturbation of nuclear lipid microdomains, localized in the inner nuclear membrane, where active chromatin is anchored. As changes of sphingomyelin species in nuclear lipid microdomains depend on neutral sphingomyelinase activity, we extended our studies to investigate whether the enzyme is modulated by daunorubicin. Indeed the drug stimulated the sphingomyelinase activity that induced reduction of saturated long chain fatty acid sphingomyelin species in nuclear lipid microdomains. Incubation of untreated-drug cells with high levels of cholesterol resulted in the inhibition of sphingomyelinase activity with increased saturated fatty acid sphingomyelin species. In daunodubicin-treated cells, incubation with cholesterol reversed the action of the drug by acting via neutral sphingomyelinase. In conclusion, we suggest that cholesterol and sphingomyelin-forming nuclear lipid microdomains are involved in the drug resistance.

## 1. Introduction

Drug resistance is the biggest obstacle for cancer treatment. Large-scale cancer drug studies provide information about the role of daunorubicin (DNR) to delay cell growth. DNR inhibits DNA synthesis, demonstrated by radiolabelled thymidine incorporation, in H35 hepatoma cells [1] and SUP-T1 lymphoma T-lymphoblast cells [2]. In HL-60 cells, DNR reduces cell viability and promotes apoptosis by modifying NF-κB; this effect is facilitated by Flavokawain B [3]. DNR-induced apoptosis of acute lymphoblastic leukemia cells is increased by the loss of the signal transducer and activator of transcription 6 (STAT6) [4]. Recently, liposomal cytarabine-daunorubicin CPX-351 has been approved as a new drug for treating acute myeloid leukemia [5]. The use of DNR combined with cytarabine and trans retinoic acid was able to induce complete remission of concomitant myelodysplasia-related acute myeloid leukemia and acute promyelocytic leukemia in a 43-year old patient [6]. A promising cancer therapy has been developed endocapsuling DNR in injectable polymeric nanocontainers [7]. The focus on DNR resistance has expanded from its origin in drug regulation to multiple biological processes of cancer [8,9]. Discovering cancer-resistance drivers and metabolites involved in the process is an interesting research area. In Breast Cancer MCF-7 Cells, P-Glycoprotein is recognized to be implicated in DNR resistance [10]. High expression of the ATP-binding cassette-multi-drug efflux protein 1 confers resistance to DNR in mucosal-associated invariant T cells [11]. In addition, altered expression of atg5, HSPA1B, collagen XIII, collagen XXAI, slug, snail, and zeb1 genes has been related to multiple drug resistance, including DNR-resistance [12]. In a previous study we found a critical role of cholesterol (CHO) and sphingomyelin (SM) in DNR resistance [2].

In particular, treatment with CHO reverses the effects of DNR in lymphoma lymphoblastic cells, by reducing neutral sphingomyelinase (nSMase) activity, increased with the drug [2]. At present, studies on the mechanisms underlying the relation between structure and function of nSMase show that the activation of the enzyme is influenced by pH, ions as Ca^2+^, Mg^2+^, and Mn^+^, phosphorylation, and anionic phospholipid-signaling molecule interaction [13]. Implication of SM metabolism in DNR-induced apoptosis has been widely described [14,15]. A majority of the current studies concentrate on finding sphingolipids that are changed in whole cancer cells with DNR, without information regarding nuclear lipids that might perturb chromatin processes. Our results on the role of nuclear lipids support the notion that SM and CHO are organized in the inner nuclear membrane to form nuclear lipid microdomains (NLM) that act as platform for active chromatin attachment [7], DNA duplication [8] and transcription [7], vitamin D-vitamin D receptor link [9], dexamethasone action on cell proliferation [10]. Considerable evidence has implicated CHO in the change of NLM in cancer cells [11]. Numerous studies have shown that the interaction CHO-SM to form microdomains or lipid rafts depends on the length of the acyl chain of SM [16,17]. Notably, our own recent study found that in NLM from hepatoma cells (H35), the fatty acids (FAs) of SM shifts from very long FAs (24:0 SM) to long FAs (16:0 SM) in comparison with normal cells, in association with an increase of proteins involved in hepatocarcinogenesis [18].

In this study, we developed a novel experimental model, which utilizes DNR, CHO, and DNR + CHO in the same cells (H35) to search for the role of NLM in drug resistance. Applying our study to hepatoma cells, we defined the perturbation of NLM induced by DNR treatment and investigated SM metabolism as a candidate pathway involved in the CHO-induced drug resistance.

## 2. Results

### 2.1. Specific Neutral Sphingomyelinase Down-Regulation with Daunorubicin Treatment

**Figure 1:**
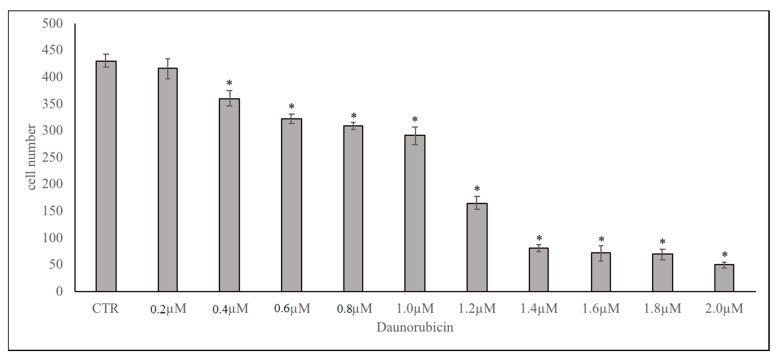
Dose-dependent effect of DNR on H35 cells: 300 × 10^3^ cells were seeded and cultured with increasing doses of DNR from 0.2 to 2.0 µM. CTR, control cells. Data indicate cell number after 24 h of culture and are expressed as the mean ± SD of three independent experiments. ∗ *p* < 0.05 versus CTR.

**Figure 2:**
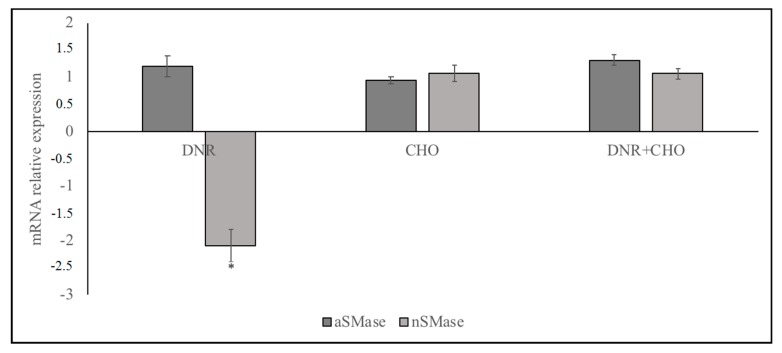
Effect of DNR, CHO, DNR–CHO on aSMase and nSMase gene expression. Cells were treated with 1 µM DNR or 800 nM CHO or 1 µM DNR + 800 nM CHO. RTqPCR analysis was performed on control (CTR) and treated cells, by using GAPDH as a housekeeping gene. On the ordinate, mRNA relative expression = mRNA of treated cells/mRNA of control cells. Data are expressed as the mean ± SD of three independent experiments performed in three PCR replicates. ∗ *p* < 0.05.

Considerable research has implicated DNR as an anti-cancer drug [1,3,4,5,6,7]. Previously, we reported that DNR delayed lymphoma cell growth and high levels of CHO induced DNR-resistance [2]. Here, we explored the underlying mechanisms of this effect considering the important relationship of CHO and SM in the nucleus, regulated by nSMase [19]. A dose-response of DNR study revealed that, by increasing the dose from 0.2 to 2 μM, the number of cells was sharply reduced for concentrations higher than 1 μM (Figure 1, Supplementary Table S1) and therefore this concentration was used for all experiments; 800 nM CHO was chosen because it corresponds to a blood condition of hypercholesterolemia [2]. Under these conditions (1 μM DNR and/or 800 nM CHO), we first set out to investigate the effect of DNR and/or CHO on aSMase and nSMase gene expression in order to exclude the involvement of aSMase. We found the down-regulation of nSMase gene expression with DNR. As can be seen, CHO alone did not change nSMase and, when combined with DNR, did not allow the DNR to act (Figure 2, Supplementary Table S1). Results showed that aSMase was not changed with all treatments (Figure 2, Supplementary Table S1).

### 2.2. Sphingomyelin Metabolism of Nuclear Lipid Microdomains as an Emerging Target of Daunorubicin in Cancer: Role of Cholesterol in Drug-Resistance

**Figure 3:**
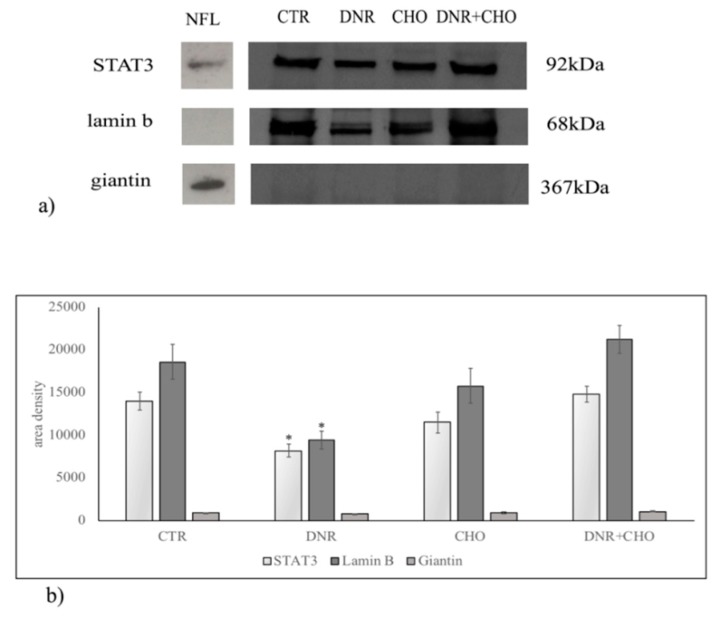
Immunoblotting analysis of nSMase, STAT3, lamin b, and giantin in nuclear lipid microdomains. For comparison, on the left, are the proteins present in nuclei free-lysates (NFL) prepared from control sample. Cells were treated with 1 µM DNR or 800 nM CHO or 1 µM DNR + 800 nM CHO. (**a**) The position of the 92 kDa for STAT3, 68 kDa for lamin b and 367 kDa for giantin was evaluated in relation to the position of molecular size standards; (**b**) the area density was quantified by densitometry scanning and analysis with Scion Image. Data represent the mean ± SD of six independent experiments. * *p* < 0.05.

**Figure 4:**
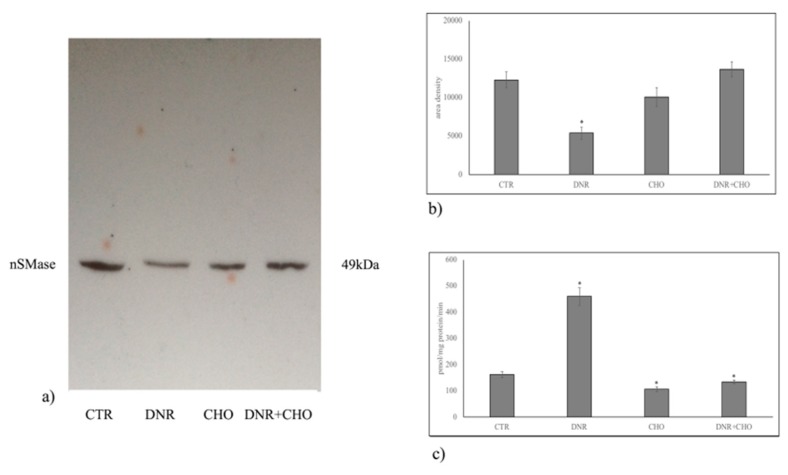
Effect of DNR, CHO, DNR–CHO on nSMase protein expression and activity in nuclear lipid microdomains purified from hepatoma cells. Cells were treated with 1 µM DNR or 800 nM CHO or 1 µM DNR + 800 nM CHO. (**a**) Immunoblotting. The position of the 49 kDa for nSMase was evaluated in relation to the position of molecular size standards; (**b**) the area density was quantified by densitometry scanning and analysis with Scion Image; (**c**) enzyme activity, data are expressed as pmol/mg protein/min. Results for b and c represent the mean ± SD of three independent experiments. * *p* < 0.05.

**Figure 5:**
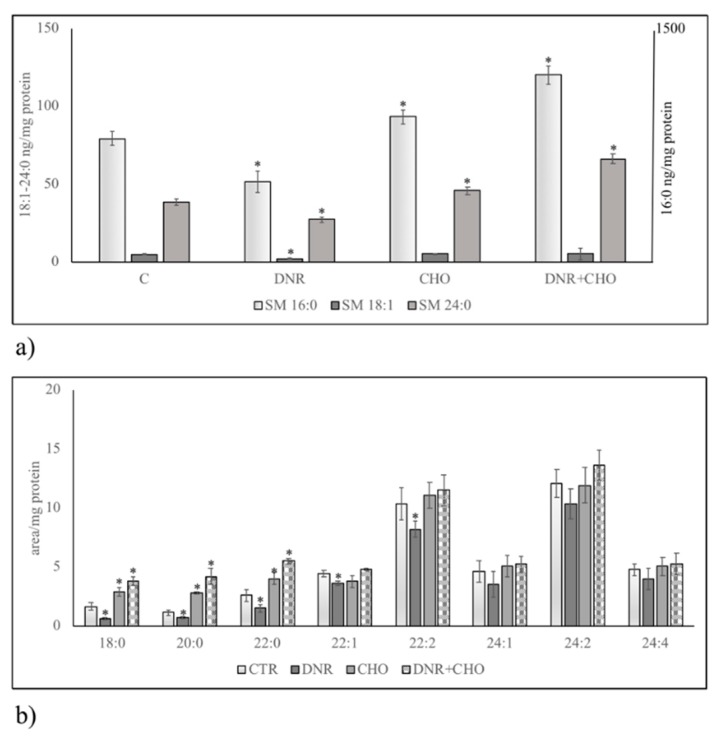
SM species in control (CTR) and DNR, CHO, DNR–CHO-treated cells in nuclear lipid microdomains purified from hepatoma cells. Cells were treated with 1 µM DNR or 800 nM CHO or 1 µM DNR + 800 nM CHO. (**a**) SM species studied by using 16:0 SM, 18:1 SM, and 24:0 SM external calibrators. Data are expressed as nmol/mg protein and represent the mean ± SD of three separate experiments. Data of SM 16:0 are referred to on the right ordinate; (**b**) SM species studied by evaluating the areas of all the peaks identified on the basis of their molecular weight. Data are expressed as area/mg protein and represent the mean ± SD of three separate experiments. * *p* < 0.05.

To clarify the role of NLM rich in CHO and SM in our experimental system, hepatoma cells were treated with DNR, CHO or DNR + CHO and NLM were purified and analyzed. First, we studied the protein expression of markers for NLM purity, such as STAT3 and lamin B, and endoplasmic membrane marker, such as giantin, in order to exclude possible contamination during extraction, as previously reported [16]. For comparison, the proteins were analysed in nuclei-free lysates (NFL), prepared from the control sample, in which the presence of STAT3 and giantin and the absence of lamin B were shown. We found both the presence of STAT3, lamin B and the absence of giantin in all samples, indicating a high level of purification of NLM (Figure 3a). Only DNR treatment induced a significant reduction of STAT3 and lamin B protein (Figure 3b, Supplementary Table S1). Then, we focused the attention on the involvement of SM metabolism in NLM as a target of DNR and on CHO-induced drug resistance. Thus, we performed analysis of the nSMase protein content in NLM. Results showed that nSMase expression was significantly repressed only with DNR treatment (Figure 4a,b, Supplementary Table S1). Consistent with the results, we assayed the enzyme activity in NLM purified from H35 cells after different treatments. Strikingly, upon DNR treatment, the nSMase activity increased strongly, and CHO or DNR–CHO treatment slightly inhibited the activity in comparison with the control sample (Figure 4c, Supplementary Table S1). Since nSMase activity modified the SM content, there was the possibility that the composition of SM species was changed in the different experimental samples. To consolidate this hypothesis, we performed UFLC-MS/MS analysis for SM species in NLM from control and treated samples, by using 16:0 SM, 18:1 SM, and 24:0 SM external calibrators. Notably, DNR reduced mainly 16:0 SM; differently, CHO and DNR + CHO increased only saturated FA SM (Figure 5a, Supplementary Table S1). In addition, to have a deeper insight into SM species, we analyzed the areas of all the peaks identified on the basis of their molecular weights. Considering the high level of SM 16:0, DNR treatment induced mainly a loss of saturated chain SM species; CHO and DNR–CHO treatment increased only long-chain saturated FAs (Figure 5b, Supplementary Table S1).

## 3. Discussion

Daunorubicin is an anticancer drug that belongs to the anthracyclines class, a well-known inducer of DNA damage through generation of reactive oxygen species [20,21]. However, the intranuclear site where the damage is induced has not been investigated. Consolidating our previous studies that hint at a role of NLM in anchoring active chromatin and in regulating duplication and transcription processes [16,17,18,19,22,23], we demonstrate that DNR induces perturbation of NLM. We found a reduction of NLM markers, such as STAT3 and lamin B, usually used as reference proteins [16,17,18,19,22,23]. Excluding a possible experimental error because the experiments were repeated many times, we suppose that the DNR induces damage of the NLM organization. Consequently, part of the marker proteins are lost during the extraction. Interestingly, despite that DNR reduces the nSMase protein content, it strongly stimulates the nSMase enzyme activity that is responsible for the reduction of SM species particularly with saturated long-chain FAs. It is difficult to explain why the nSMase protein is reduced. There are two possibilities: (1) the demonstrated down-regulation of the nSMase gene expression is responsible for the reduction of the enzyme in different cellular sites and, therefore, also in NLM; (2) the protein is lost together with STAT3 and lamin B. Furthermore, it is possible to suggest another mechanism of action of the DNR. This study adds to an emerging literature implicating the role of anthracyclines in cancer via SM metabolism [24]. Our results show that the DNR, by activating nSMase, reduces mainly saturated long-chain FA SM species. In H35 cells, NLMs are rich in SM species with saturated long chains [18]. It is known that CHO prefers saturated SM to establish more favorable entropic interactions essential for the formation of NLM [25]. Therefore, it cannot be ruled out that DNR, by reducing the SM species, destructures NLM with loss of STAT3 and lamin B. These different hypotheses open up two scenarios: (1) the DNR first destroys NLM and then activates nSMase or (2) the DNR acts directly on nSMase activity and consequently destroys the NLMs. The latter mechanism would explain why CHO, by reducing nSMase activity and enriching the NLM of saturated long-chain FA SM, has an opposite action to that of DNR, if added alone. If the CHO is added together with the DNR, it does not allow the drug to act. It is important to consider that the dose used for the experiments corresponds to a condition of hypercholesterolemia [2]. We demonstrated that cancer cells incorporate CHO by inducing severe hypocholesterolemia. CHO entering the cancer cells stimulates their proliferation [2]. A systematic review on statins and colon cancer highlights that lowering blood cholesterol level reduces colorectal carcinogenesis [26]. The role of CHO in cancer is widely demonstrated even if the studies on mechanism of action are still subject to discussion. As more studies begin to elucidate the role of CHO inside the cancer cells, we propose to consider that hypercholesterolemia might induce drug resistance by changing the lipids inside the nucleus, in NLM. The study suggests that hypercholesterolemia can be harmful in a patient with cancer as responsible for drug-resistance.

## 4. Materials and Methods

### 4.1. Materials

H35 hepatoma cells were obtained from the European Collection of Animal Cell Cultures (Salisbury, UK). DMEM, bovine serum albumin (BSA), dithiothreitol (DTT), fetal bovine serum (FBS), methanol, 3-(4,5-dimethyl-thiazol-2-yl)-2,5-diphenyltetrazolium bromide, 2-propanol, metyl-tert-butyl ether, formic acid, chloroform, DNR and CHO were obtained from Sigma-Aldrich (St. Louis, MO, USA). Lipid standards 16:0 SM, 18:1 SM, 24:0 SM were purchased from Avanti (Avanti Polar, Alabaster, AL, USA). Anti-giantin, anti-signal transducer and activator of transcription 3 (STAT3), anti-nSMase, and anti-aSMase antibodies were obtained from Santa Cruz Biotechnology (Santa Cruz, CA, USA); anti-lamin B was obtained from Oncogene (Boston, MA, USA).

### 4.2. Cell Culture

H35 hepatoma cells were grown as previously reported [18]. For dose-dependent effect of DNR study, cells were seeded at 10 × 10^4^/mL concentration, incubated with increasing doses of DNR from 0.2 to 2.0 µM and counted after 24 h. For all experiments, four lots of the cells at 10 × 10^6^ concentration were prepared: the control sample (C) without DNR and CHO, the experimental sample with 800 nM CHO [11] or with 1 µM DNR [2] or with 800 nM CHO + 1 µM DNR. After 24 h of culture, a small amount of the cells was used for aSMase and nSMase gene expression and the remaining part NLM purification.

### 4.3. Nuclei-Free Lysates

The cells were washed twice with phosphate-buffered saline (PBS) and centrifuged at 800× *g* for 10 min. The resulting pellet was suspended in hypotonic buffer (1.5 M sucrose, 3 mM CaCl_2_, 2 mM magnesium acetate, 0.5 mM DTT, 1 mM phenylmethylsulfonylfluoride (PMSF), 3 mM Tris-HCl, pH 8.0 (1 mL/10^6^ cells)), gently homogenized using a tight-fitting Teflon-glass homogenizer and centrifuged at 500× *g* for 30 min at 4 °C for nuclei-free lysates (NFL) preparation, as previously reported [27]

### 4.4. Purification of Nuclear Lipid Microdomains

NLMs were purified H35 cell nuclei as previously reported [10]. Briefly, the homogenized cells were treated with 1% Triton X-100 in hypotonic buffer (1.5 M sucrose, 3 mM CaCl_2_, 2 mM magnesium acetate, 0.5 mM DTT, 1 mM PMSF, 3 mM Tris-HCl, pH 8.0) 0.5:1 vol/vol and then treated with 1 M sucrose, followed by centrifugation to isolate whole nuclei. The removal of the external nuclear membranes with preservation of the inner nuclear membranes, was obtained by a 10 mM Tris solution containing 2.5 mM MgCl_2_, 0.5 mM PMSF, and 1% Triton X-100. NLMs were extracted from inner nuclear membrane with a cushion of 80% sucrose with a gradient of 15–40% sucrose on top. After overnight centrifugation, the gradient had five 2-mL fractions plus two 1-mL floating fractions. The latter fractions were carefully collected, diluted five times with 25 mM HEPES-HCl solution containing 150 mM NaCl, and centrifuged at 100,000× *g* for 2 h to obtain a pellet of NLM.

### 4.5. Reverse Transcription Quantitative PCR (RT-qPCR)

After 24 h of culture, cells were used for total RNA extraction by using RNAqueous^®^-4PCR kit and for RT-qPCR performed by using Master Mix TaqMan^®^Gene Expression with a 7.500 RT-PCR instrument (Applied Biosystems, Monza, Italy), as previously reported [17]. The targeted genes were: SM phosphodiesterase 1 (SMPD1, Hs03679347_g1) and SM phosphodiesterase 4 (SMPD4, Hs04187047_g1). Glyceraldehyde-3-phosphate dehydrogenase (GAPDH, Hs99999905_m1) was used as a housekeeping gene. Relative quantification of SMPD1 and SMPD4 genes, after 24 h of drug treatment, was calculated in comparison to the relative controls and normalized to the housekeeping gene GAPDH, using the 2^−ΔΔ^*^C^*^t^ method.

### 4.6. Protein Content

Total protein concentration was determined spectrophotometrically at 750 nm by using bovine BSA as a standard, as previously reported [16].

### 4.7. Electrophoresis and Western Blot Analysis

Proteins (∼30 μg) underwent SDS-PAGE electrophoresis in 12% polyacrylamide slab gel for nSMase, STAT3, lamin B and 8% for giantin, according as previously reported [16]. The transfer of protein was carried out into nitrocellulose in 90 min, the membranes were blocked for 30 min with 5% nonfat dry milk in PBS (pH 7.5), and incubated overnight at 4 °C with primary antibodies. The blots were treated with horseradish-conjugated secondary antibodies for 90 min. Visualization was performed with the Enhanced Chemiluminescence (ECL) kit from Amersham (GE Healthcare Europe GmbH, Milano, Italy). The position of the protein was indicated in relation to the position of molecular size standards. The area density was evaluated by densitometry scanning and analysis with Scion Image software (https://scion-image.software.informer.com/4.0/).

### 4.8. nSMase Activity Assay

H35 cells were suspended in 0.1% NP-40 detergent in PBS, sonicated for 30 s on ice at 20 watt, kept on ice for 30 min and centrifuged at 16,000× *g* for 10 min. The supernatants were used for nSMase assay. The enzyme activity was assayed with Amplex Red Sphingomyelinase assay kit (Invitrogen, Monza, Italy) by using 60 μg proteins/10 µL Tris-MgCl_2_ pH 7.4. The fluorescence was measured with FLUOstar Optima fluorimeter (BMG Labtech, Monza, Italy), by using the filter set of 360 nm excitation, and 460 nm emission.

### 4.9. Lipid Extraction and UFLC MS/MS Analysis

Lipid extraction and UFLC MS/MS analysis were performed as reported by Lazzarini et al. (2015) [18] The 16:0 SM, 18:1 SM, 24:0 SM standards were prepared and dissolved in chloroform/methanol (9:1 vol/vol) at 10 μg/mL final concentration. The stock solutions were stored at −20 °C. Working calibrators were prepared by diluting stock solutions with methanol to 500:0, 250:0, 100:0, and 50:0 ng/mL final concentrations. Twenty microliters of standards or lipids extracted from serum was injected after purification with specific nylon filters (0.2 μm). Analyses were carried out by using the Ultra Performance Liquid Chromatography system tandem mass spectrometer (Applied Biosystems, Italy). The samples were separated on a Phenomenex Kinetex phenyl-hexyl 100 A column (50 × 4.60-mm diameter, 2.6-μm particle diameter) with a precolumn security guard Phenomenex ULTRA phenyl-hexyl 4.6. For SM, column temperature was set at 50 °C and flow rate to 0.9 mL/min. Solvent A was 1% formic acid; solvent B was 100% isopropanol containing 0.1% formic acid. The run was performed for 3 min in 50% solvent B and then along a gradient to reach 100% solvent B in 5 min. The system needed to be reconditioned for 5 min with 50% solvent B before the next injection. The SM species were identified by using positive turbo-ion spray and modality multipole-reaction monitoring [18].

### 4.10. Statistical Analysis

Data were expressed as means ± SD and their significance was checked by Student’s *t*-test. * *p* < 0.05 versus control samples (CTR).

## Supplementary Materials

Supplementary materials can be found at http://www.mdpi.com/1422-0067/19/11/3424/s1.
