# Supplementary figures and images for: Nuclear Lipid Microdomains Regulate Daunorubicin Resistance in Hepatoma Cells
Source: Int J Mol Sci. 2018 Nov 1;19(11):3424. doi: 10.3390/ijms19113424 (PMC6274808; doi:10.3390/ijms19113424)

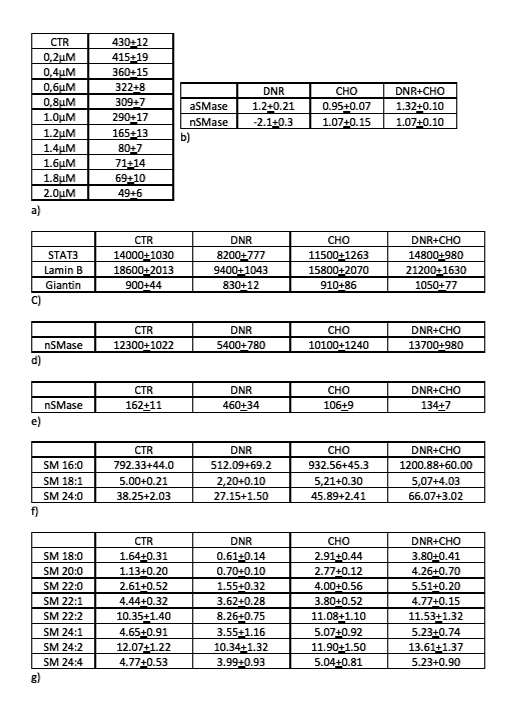

Supplement: Supplementary file 1 [file ijms-19-03424-s001.zip › ijms-368668-SI.tiff]
